# Combined biochar and DMPP reduce N_2_O emissions in wheat crops via microbial community modulation

**DOI:** 10.3389/fpls.2025.1647453

**Published:** 2025-10-01

**Authors:** Haizhong Wu, Dengxiao Zhang, Xiaobo Shen, Guozhen Ma, Qingsong Yuan, Hongjing Zhao, Shiliang Liu, Xiaolei Jie, Daichang Wang

**Affiliations:** ^1^ College of Resources and Environment, Henan Agricultural University, Zhengzhou, China; ^2^ College of Geography and Planning, Chizhou University, Chizhou, China; ^3^ Key Laboratory of Arable Land Quality Conservation in the Huanghuaihai Plain, Ministry of Agriculture and Rural Affairs, Zhengzhou, China

**Keywords:** N_2_O, biochar, DMPP, AOB, nirK, nosZ

## Abstract

Delayed nitrogen (N) application increases N use efficiency in a broadacre cropping system. However, its effect on N_2_O emissions and the underlying microbial mechanisms remains poorly understood. A field-plot experiment was carried out to examine the effects of biochar and a nitrification inhibitor (DMPP) on soil N_2_O emissions with six treatments: without N application (control), optimal N application (ON), farmer conventional N application (FN), biochar + ON (ONB), DMPP + ON (OND), and biochar + OND (ONDB). In comparison to the ON treatments, cumulative N_2_O emissions from the OND and ONDB treatments were significantly reduced by 32% and 38%, respectively, whereas emissions from the FN and ONB treatments exhibited increases of 38% and 4%, respectively. N application or biochar amendment increased the abundance of AOA and AOB, whereas DMPP amendment led to a reduction in AOB abundance. The OND and ONDB treatments enhanced the relative proportion of *Nitrospira* in the AOB community. The ONB treatment altered the most dominant genus of *nirS* and *nosZ* communities. Correlation analysis revealed that AOB, *nirK*, and *nirK/nosZ* were the predominant microorganism communities influencing soil N_2_O emissions. Random forest analysis identified *Nitrospira* in AOB communities, *Cronobacter* in *nirK*-containing communities, and *Ramlibacter* and *Methylobacillus* in the *nosZ*-containing community as key microbial taxa contributing to N_2_O emissions. We propose that the ONBD treatment provides dual advantages by reducing N_2_O emissions and enhancing N use efficiency under the delayed N application regime.

## 1 Introduction

Nitrous oxide (N_2_O) contributes approximately 7% to the overall global warming phenomenon (Li et al., 2020). Since 1975, the atmospheric concentration of N_2_O has risen by 23%, reaching to the current level of 332 ppb, the highest concentration documented in more than 800,000 years (IPCC, 2023). Emissions of N_2_O from agricultural systems are largely attributed to the application of nitrogen (N) fertilizers, resulting in the annual release of more than 4 Tg N_2_O-N (Yu et al., 2023). To address the escalating food demands of the world population, the quantity of synthetic N fertilizer applied in crop production continues to rise (Aryal et al., 2022). Urea, a globally prevalent synthetic N fertilizer, exhibits suboptimal utilization efficiency, resulting in significant N loss (approximately 40%) through various pathways (Liu et al., 2010), such as gaseous N emissions (e.g., N_2_O, NO) and nitrate-nitrogen (NO_3_^−^-N) (Klimczyk et al., 2021). From a sustainable development perspective, agricultural modernization must achieve precise N management to ensure food security and mitigate climate change.

The conventional approach to minimizing N_2_O emissions in agricultural production involves optimizing N application regimes and reducing the overall amount of N applied (Hartmann et al., 2015). Several studies have assessed the effects of various N application management strategies on mitigating N_2_O emissions, including deep application of N fertilizer (Wu et al., 2021), integration of urea and organic fertilizers (Wei et al., 2024), optimization of agricultural practices (Ashiq et al., 2021), and advances in irrigation techniques (Zhong et al., 2021). However, the impact of the timing of crop N application on N_2_O emissions has been largely overlooked in recent decades. Improvement of N use efficiency (NUE) cannot be accomplished instantly owing to the complexity of N uptake and utilization by crops (Qiao et al., 2015). Premature application of N fails to consider appropriate matching of N supply and N demand of winter wheat (Cui et al., 2010), resulting in significant N loss (Ding et al., 2010). Indeed, the N requirements of winter wheat differ among developmental stages, and soil N mineralization can effectively meet the early N demands of wheat (Sylvester-Bradley et al., 2001). Engel et al. (2017) considered that deferral of N application until spring was more appropriate to fulfill the N requirements of winter wheat. Application of a basic N fertilizer during the tillering stage of winter wheat has been shown to significantly enhance NUE (Wallace et al., 2020). Similarly, Yao et al. (2024) reported that delayed application of fertilizers is beneficial for increasing wheat yield. Thus, delaying N application until spring and applying N fertilizer as a topdressing during the critical phase for N demand by winter wheat may represent a viable approach to mitigate N_2_O emissions. The combined application of N fertilizers and synergistic agents represents a robust strategy for mitigating yield losses in crops caused by diminished N application (Huang et al., 2019). Nitrification inhibitors (NIs), serving as soil synergists, exhibit remarkable advantages in mitigating N_2_O emissions and reducing N losses (Liu C. et al., 2021; Dawar et al., 2021). Notably, 3,4-dimethylpyrazole phosphate (DMPP) has been shown to effectively reduce N_2_O emissions and NO_3_^−^-N leaching in agricultural systems. As a sustainable material for soil improvement, the potential environmental benefits of biochar are being increasingly validated. In the North China Plain, biochar amendment at a rate of 15 t ha^−1^ represents an optimal strategy for achieving high grain yields while substantially reducing N fertilizer inputs (Huang et al., 2022). He et al. (2018) reported that the application of biochar at 15 t ha^−1^ markedly reduced N_2_O emissions in wheat fields by 49.69%. Both positive and negative impacts on N_2_O emissions of N fertilizer applied in conjunction with synergistic agents have been reported (An et al., 2022; Duan et al., 2019; Verhoeven and Six, 2014). The inconsistent findings present a significant challenge to the predictive analysis of the impact of synergistic agents on N_2_O emissions. To date, it remains unclear how do DMPP and biochar interact under delayed N regimes to shape microbial N_2_O pathways.

Production of N_2_O from agricultural soils is primarily driven by microbial involvement in the nitrification and denitrification processes (Pihlatie et al., 2004). The investigation of nitrifying and denitrifying microorganisms provides vital insights into the mechanisms that govern N_2_O emission (Wang et al., 2024). The nitrification pathway contributes to N_2_O emissions from dryland soils (Shaaban, 2024). Nitrification plays a crucial role in mediating N_2_O emissions, particularly through ammonia oxidation and nitrifier denitrification, which are predominantly regulated by ammonia-oxidizing microorganisms (Martens-Habbena et al., 2015). An increasing body of evidence indicates that DMPP mitigates N_2_O emissions primarily by inhibiting nitrification, particularly through suppression of AOA and AOB activities. Biochar application significantly enhances N_2_O emission, which is attributable to biochar-stimulated increase in the activity of AOB and AOA (Lin et al., 2017). Research on denitrifying bacteria is crucial to elucidate the mechanisms of N_2_O emission under various fertilization practices (Huang et al., 2021). The microbial genes *nirS*, *nirK*, and *nosZ* play pivotal roles in denitrification (Shen et al., 2021; Li et al., 2020). The reduction of nitrite to NO, primarily mediated by *nirS* and *nirK*, is a rate-limiting step in the denitrification pathway (Liang et al., 2021). The transformation of N_2_O to dinitrogen is predominantly catalyzed by a N_2_O reductase encoded by *nosZ* (Shaaban et al., 2023). However, the impacts of DMPP and biochar application on N_2_O generation mediated by denitrifying bacteria in dryland soils remain unclear. In addition, the response of soil N_2_O emissions to DMPP and biochar application, together with microbe-mediated mechanisms of N_2_O production in dryland soils, under delayed N application is poorly understood.

The North China Plain is an important dryland agricultural region in China and accounts for 66% of the total wheat production area in the country. More than 70% of the farmland is subjected to excessive N application, with the annual input of synthetic N at 550–600 kg N ha^−1^ (Song et al., 2018). The region has emerged as a ‘hot spot’ for N_2_O emissions in China. Given this context, we investigated the effects of combined application of synergists on N_2_O emission and its underlying microbial mechanisms under a delayed N application regime. We hypothesized that: 1) delayed N application may effectively reduce soil N_2_O emissions; 2) the combined application of biochar and DMPP represents the most effective strategy for mitigating N_2_O emissions; and 3) the inhibitory mechanism of the combination of biochar and DMPP on N_2_O emissions via microbial community modulation. The aims of the field-plot experiment were 1) to elucidate the impact of N fertilizer application in combination with synergists on N_2_O emission under delayed N application, and 2) to investigate the influence of DMPP and biochar on the abundance and diversity of microbial functional genes associated with N_2_O emission.

## 2 Materials and methods

### 2.1 Field site

**Figure 1:**
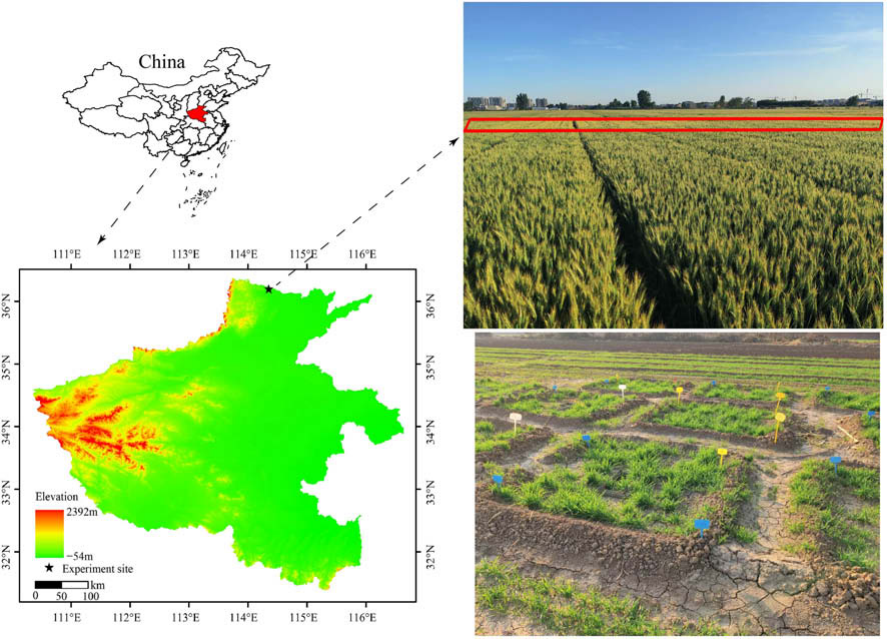
Map of the study area.

The field-plot experiment was carried out in 2022–2024 at Anyang (36°11’51’’N, 114°20’56’’E), Henan Province, China (**Figure 1**). The soil type is classified as a Fluvisols. The study site has an average elevation of 84.3 m above sea level, and the mean annual temperature and rainfall is 14°C and 557 mm, respectively. The soil pH was 7.57, and the contents of soil organic carbon (SOC), total N (TN), available phosphorus, and available potassium were 11.22 g kg^−1^, 1.09 g kg^−1^, 12.9 mg kg^−1^, and 89.2 mg kg^−1^, respectively.

### 2.2 Experimental design

Six treatments were applied in the delayed N application experiment during the 2022-2023 winter wheat growing season: a control group without N fertilizer application (CK); two rates of N fertilizer application, namely, 180 kg N ha^−1^ (optimal N application; ON) and 270 kg N ha^−1^ (farmer conventional N application; FN); ON + biochar at the rate of 15 t ha^−1^ (ONB); ON + DMPP (OND); and ON + biochar + DMPP (ONDB). Five treatments were applied in the normal N application experiment during the 2023-2024 winter wheat growing season. The experimental treatments included CK, ON, ONB, OND, and ONDB. It is well established that elevated N application rates lead to increased N_2_O emissions and higher emission factors, the experimental results from the first wheat-growing season fully support this conclusion. This study focuses on the environmental effects resulting from the integration of optimal N application (ON) with biochar or DMPP. Therefore, the FN treatment was excluded from the normal N application regime.

Urea was utilized as the N fertilizer applied in two distinct phases: 60% of the N fertilizer was applied during the first fertilization, and the remaining 40% was applied as topdressing (**Table 1**). In addition, phosphate and potassium fertilizers, together with biochar, were applied on October 20, 2022 and October 16, 2023. Biochar was prepared from corn stalks at 450°C. The biochar C and N contents were 507 g kg^−1^ and 2.1 g kg^−1^, respectively, and the pH was 9.7. Each experimental plot has an area of 2 m × 2 m, with three replicates per treatment. Wheat seeds were sown on October 25, 2022, and October 16, 2023, while the mature grains were harvested on June 10, 2023 and June 4, 2024, respectively.

**Table 1:** Experimental treatments of wheat under two N application regimes.

Fertilization regime	Treatment	Urea (kg N ha^−1^)	Phosphate (kg P_2_O_5_ ha^−1^)	Potassium (kg K_2_O ha^−1^)	First N application time	Topdressing N application time	Note
Delayed N application	CK	0	60	45			
	ON	180	60	45			
	CN	270	60	45	February 8, 2023	April 17, 2023	
	ONB	180	60	45			Biochar: 15 kg ha^−1^
	OND	180	60	45			DNPP: 1.8 kg ha^−1^ (1% of urea-N)
Normal N application	ONDB	180	60	45			Biochar: 15 kg ha^−1^, DNPP: 1.8 kg ha^−1^
	CK	0	60	45			
	ON	180	60	45			
	ONB	180	60	45	October 16, 2023	March 10, 2024	Biochar: 15 kg ha^−1^
	OND	180	60	45			DNPP: 1.8 kg ha^−1^
	ONDB	180	60	45			Biochar: 15 kg ha^−1^, DNPP: 1.8 kg ha^−1^

### 2.3 N_2_O gas sampling and measurements

Nitrous oxide gas was collected in a sealed chamber, following the methodology described by Wu et al. (2024). Eighteen static opaque chamber bottoms were inserted into the soil within the study plot at 8 cm depth until the harvest of winter wheat. The static chambers were equipped with an electric fan and a thermometer on top. Gas samples were extracted from the chamber at four time points (0, 15, 30, and 45 min) using a 50 ml plastic syringe following its closure. Simultaneously, the temperature inside the static chambers was recorded. The electric fan operated continuously throughout the sampling process to maintain air homogeneity within the enclosed space. A total of 72 gas samples were collected each sampling day over a continuous 7-day period following N application; thereafter, the sampling was conducted at 7- to 10-day intervals.

N_2_O flux was determined using a GC-2010 Plus gas chromatograph. The emission flux of N_2_O (*f*) was calculated with the following formula:


f=ρ×(V/A)×(ΔC/ΔT)×273/(273+T) 

where ρ (kg m^−3^) is the N_2_O density, *V* is the volume of the sealing chamber (m^3^), *A* is the bottom area of the chamber (m^2^), Δ*C*/Δ*T* (μL L^−1^ h^−1^) is the temporal variation in N_2_O concentration in the sealed chamber, and *T* (C) is the mean temperature inside the chamber. The cumulative emission of nitrous oxide (CE-N_2_O) was estimated by employing linear interpolation of N_2_O flux and time (Allen et al., 2010).

The N_2_O emission factor was calculated as follows:


$$\text{EF}=({\text{CE}}_{\text{N fertilizer}}-{\text{CE}}_{\text{no N fertilizer}}\text{)}/{\text{N}}_{\text{input}}$$

where CE_N fertilizer_ and CE_no N fertilizer_ represents CE-N_2_O from treatments with N application and without, respectively. N_input_ represents the amount of N fertilizer applied.

### 2.4 Analysis of soil physicochemical parameters

Fresh soil samples were collected subsequent to gas sampling for determination of the ammonia-N (NH_4_^+^-N) and Nitrate-N (NO_3_^−^-N) contents, which were extracted using 2 M L^−1^ KCl solution and subsequently determined with a flow analyzer. Soil samples collected at harvest were used to determine physicochemical parameters. Dissolved organic N (DON) was measured by subtracting NH_4_^+^-N and NO_3_^−^-N from the total soluble N (TSN) content. Soil bulk density, pH, TSN, SOC), and TN were determined following soil agricultural chemistry analysis (Lu, 2000). Each sample is analyzed in duplicate, and the relative deviation between the duplicate samples must not exceed 5%. The soil water-filled pore space (WFPS) was determined as follows.


$$WFPS=\theta v/\left(1-/2.65\right)$$

Where, θv represents the volumetric water content, ρ represents for soil bulk density.

### 2.5 DNA extraction and qPCR

Total DNA from 0.5 g soil samples was extract by using the E.Z.N.A.^®^ Soil DNA Kit (Omega Bio-Tek, USA). The purified DNA was stored at −80°C until analysis. Gene copy numbers were determined using fluorescent qPCR (ABI 7500, USA) following protocols described by Huang. Information on the primers used and the parameters for the qPCR reactions are presented in **Supplementary Table S1**.

### 2.6 Microbial diversity detection and taxonomic analysis

The qPCR products were identified, purified, and quantified using 2% agarose gel electrophoresis, the AxyPrep DNA Gel Extraction Kit, and a Quantus™ Fluorometer, respectively. The NEXTFLEX Rapid DNA-Seq Kit was used to construct a DNA library, which was sequenced using an Illumina platform (NovaSeq PE250). The initial sequences were subsequently refined and concatenated to yield high-quality sequences. The UPARSE software was employed for clustering of operational taxonomic units (OTUs), following the clustering protocols and methodologies outlined by Bi et al. (2023). The RDP Classifier was used to annotate the species classification of the sequences, and the classification information for each OTU was derived by comparison with the Silva 16S rRNA database. The UCLUST algorithm was used for further taxonomic analysis of the representative OTU sequences.

### 2.7 Statistical analysis

The data were analyzed statistically with SPSS version 25.0. ANOVA was employed to assess the significance of differences among the indexes, *post hoc* tests were executed using the LSD, with a significance threshold of *P < 0.05*. Redundancy analysis was conducted using canoco 5.0 to examine the relationships between soil indicators and microbial communities. Correlation analysis was conducted using Origin 2021 software. A random forest analysis was conducted to identify microbial genera that significantly influence N_2_O emission using the ‘rfPermute’ package for R.

## 3 Results

### 3.1 N_2_O flux

**Figure 2:**
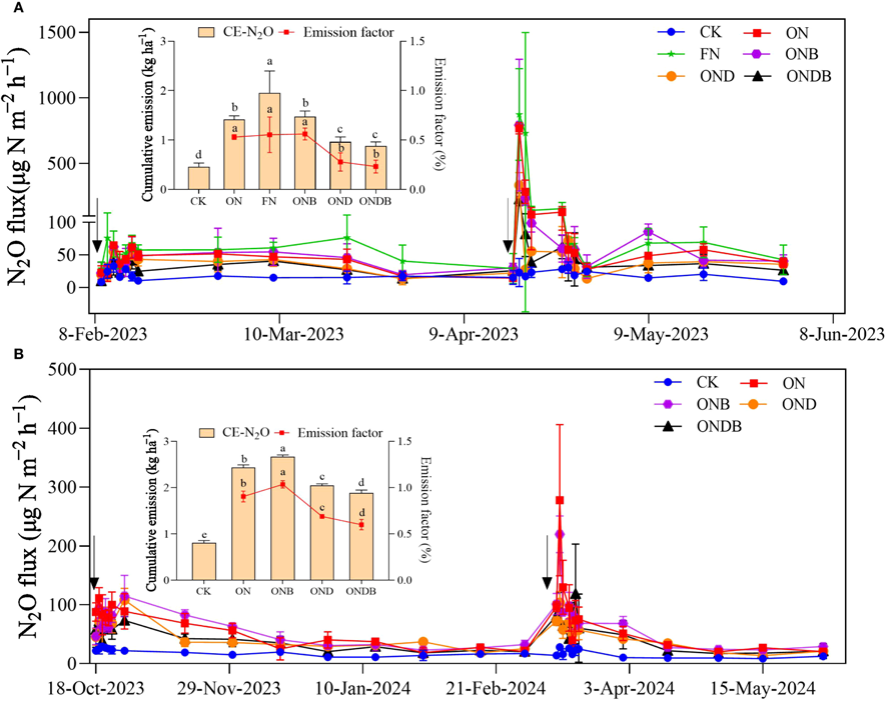
N_2_O emissions under delayed N application (2022–2023 wheat growing season) **(A)** and Normal N application (2023–2024 wheat growing season) **(B)**.The black arrows indicate the time points at which N fertilizer was applied. CK, control; ON, 180 kg N ha^−1^; FN, 270 kg N ha^−1^; ONB, ON + biochar;OND, ON + DMPP; ONDB, ONB + biochar. Different letters indicate statistically significant differences among treatments (*P* < 0.05).

The temporal dynamics of N_2_O emission showed discernible fluctuations. N fertilizer application greatly stimulated soil N_2_O emission. A distinct difference in soil N_2_O emissions between the first and second N applications was observed (**Figure 2**). The N_2_O flux increased markedly following the second N application. The N application treatments exhibited significantly higher N_2_O emissions compared with those of the CK. Under the delayed N application regime, the highest CE-N_2_O (1.96 kg ha^−1^) was observed under the FN treatment. The ONB treatment (1.47 kg ha^−1^) led to a slightly higher CE-N_2_O than in the ON treatment (1.42 kg ha^−1^) (**Figure 2A**). Addition of DMPP resulted in significant reduction of N_2_O emissions; the OND and ONDB treatments exhibited reductions in CE-N_2_O of 32% and 38%, respectively, compared with the CE-N_2_O of the ON treatment. The N_2_O emission factors for different treatments under the delayed N application regime ranged from 0.23% to 0.56%, which were substantially lower than the factors ranging from 0.60% to 1.03% under the normal N application regime (**Figure 2B**). These results indicate that implementing a delayed N application strategy can effectively mitigate N_2_O emissions.

### 3.2 Variation in soil characteristics

**Figure 3:**
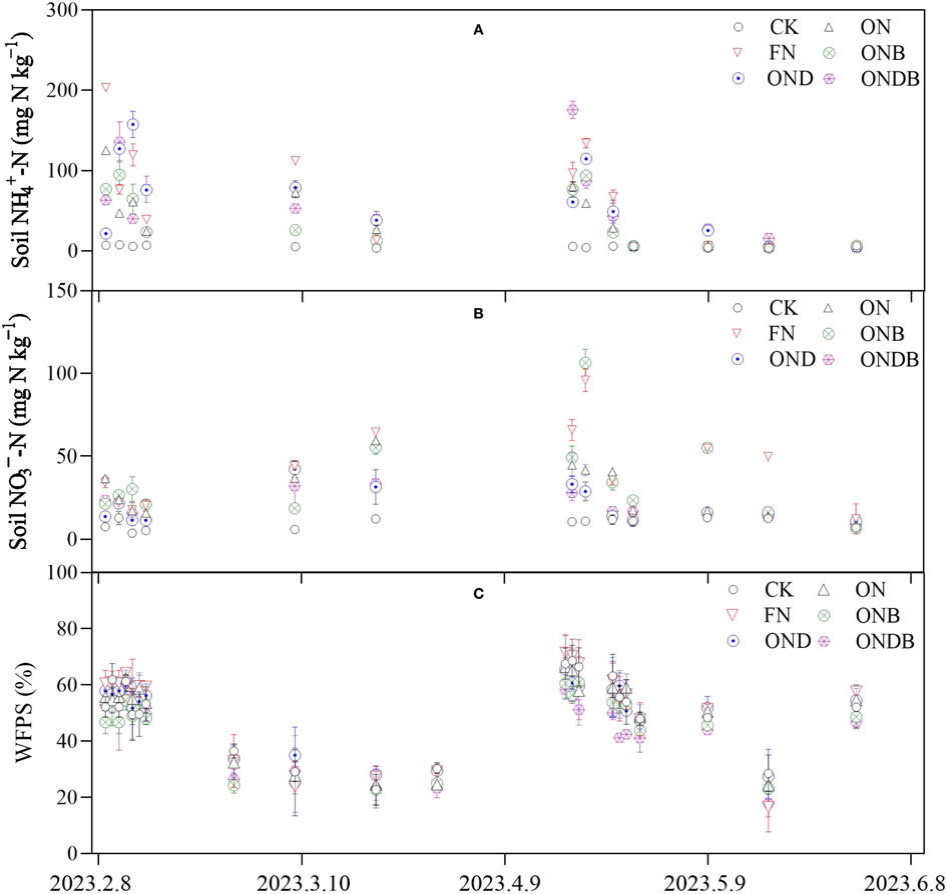
Temporal variations of NH_4_^+^-N **(A)**, nitrate (NO_3_^−^-N) **(B)**, and WFPS **(C)** of soil.

Soil NO_3_^−^-N and NH_4_^+^-N contents were significantly increased following application of N. The NH_4_^+^-N content under the ONB treatment was lower than the ON treatment following N fertilization. The NO_3_^−^-N content in response to DMPP application (the OND and ONDB treatments) was comparatively low (**Figures 3A, B**). The WFPS ranged between 16.58% and 71.46%, exhibiting similar tendencies under the different treatments (**Figure 3C**). According to the average inorganic-N content of the soil from the initial N application until the wheat harvesting period, the FN treatment resulted in the highest NH_4_^+^-N and NO_3_^−^-N contents (**Table 2**). The ONB treatment decreased NH_4_^+^-N content and significantly increased NO_3_^−^-N content, whereas treatment with DMPP (OND and ONDB) led to a significant increase in NH_4_^+^-N content and a significant decrease in NO_3_^−^-N content. The NO_3_^−^-N content differed significantly among the treatments, except for OND and ONDB. Biochar amendment significantly enhanced the soil pH and SOC content, compared with the ON treatment; the ONB and ONDB treatments increased pH by 3% and 5%, respectively, and SOC by 7% and 8%, respectively. Application of DMPP led to slight, but non-significant, increases in soil SOC and pH. The soil DON concentration increased markedly with increase in the N application rate. The ONB and ONDB treatments slightly enhanced the soil DON concentration, whereas the OND treatment had the opposite effect.

**Table 2:** Soil properties under different treatments following wheat harvest.

Treatment	Ph	BD	TN (g/kg)	SOC (g/kg)	NH_4_^+^-N (mg/kg)	NO_3_^−^-N (mg/kg)	DON (mg/kg)
CK	7.67 ± 0.02c	1.35 ± 0.05a	1.08 ± 0.01a	11.65 ± 0.44b	5.49 ± 0.35d	9.63 ± 0.01e	11.07 ± 1.39c
ON	7.58 ± 0.04d	1.38 ± 0.04a	1.11 ± 0.00a	12.47 ± 0.08ab	42.06 ± 0.43c	28.85 ± 1.36c	16.82 ± 1.36bc
FN	7.57 ± 0.03d	1.39 ± 0.02a	1.09 ± 0.01a	12.02 ± 0.42b	67.9 ± 1.52a	40.96 ± 1.44a	28.25 ± 1.22a
ONB	7.78 ± 0.04b	1.21 ± 0.08b	1.14 ± 0.01a	13.17 ± 0.92a	39.52 ± 3.40c	35.78 ± 1.25b	25.99 ± 0.11b
OND	7.71 ± 0.03c	1.41 ± 0.05a	1.16 ± 0.02a	12.54 ± 0.28ab	58.86 ± 1.71b	20.16 ± 1.33d	19.02 ± 0.90c
ONDB	7.93 ± 0.01a	1.26 ± 0.01b	1.19 ± 0.01a	13.48 ± 0.74a	59.19 ± 0.87b	20.98 ± 0.51d	25.85 ± 4.56c

Different lowercase letters in the same column indicate significant differences.

### 3.3 Abundance of N functional genes

Ammonia-oxidizing and denitrifying bacteria exhibited distinct variation in abundance among the treatments (**Table 3**), as indicated by the copy numbers of microbial functional genes. The CK, ON, and FN treatments exhibited significant elevation in AOB gene copy numbers with increasing N input, whereas no notable differences in AOA were observed. In comparison with the ON treatment, the ONB treatment markedly enhanced the abundance of AOB gene copies, whereas ONDB treatment had the most pronounced effect in reducing AOB gene copy numbers. The ONB treatment significantly enhanced the AOA abundance, whereas the OND and ONDB treatments had no significant effect. The ratio of AOA to AOB gene copy numbers was smallest under the ON treatment (0.35) and largest under the ONB treatment (0.69). The quantity of *nirS*, *nirK*, and *nosZ* gene copies declined with increase in the N application rate. Relative to the ON treatment, the ONB treatment substantially increased the *nirS* abundance, whereas the OND treatment significantly increased the abundance of *nirK* (**Table 3**). The highest number of gene copies was observed for *nirK*, whereas the lowest number of copies detected was for *nirZ*. The nirK/nosZ ratio varied between 1.27 and 1.53, with no statistically significant differences among treatments detected.

**Table 3:** The gene copy numbers of *amoA* and denitrification-related functional genes.

Treatment	AOB (10^6^ copies/g)	AOA (10^6^ copies/g)	AOA/AOB	*Nirk* (10^6^ copies/g)	*Nirs* (10^6^ copies/g)	*Nosz* (10^6^ copies/g)	*Nirk/nosz*
CK	19.54 ± 0.31c	9.44 ± 0.36b	0.48 ± 0.02ab	63.97 ± 3.46ab	5.48 ± 0.91b	44.56 ± 9.22ab	1.46 ± 0.21a
ON	25.89 ± 4.42b	8.82 ± 2.13b	0.35 ± 0.12b	51.65 ± 2.58cd	4.72 ± 0.82bc	40.57 ± 0.69ab	1.27 ± 0.04a
FN	32.54 ± 1.59a	13.56 ± 1.07b	0.42 ± 0.05b	41.81 ± 8.50e	4.16 ± 0.17c	32.53 ± 8.82b	1.32 ± 0.28a
ONB	32.11 ± 3.08a	22.13 ± 4.7a	0.69 ± 0.13a	43.4 ± 7.54de	6.93 ± 0.13a	32.5 ± 6.94b	1.34 ± 0.06a
OND	21.24 ± 5.28bc	13.59 ± 0.9b	0.67 ± 0.19a	71.99 ± 1.32a	4.62 ± 0.15bc	47.49 ± 7.07a	1.53 ± 0.19a
ONDB	17.26 ± 3.12c	9.28 ± 3.65b	0.53 ± 0.14ab	56.46 ± 4.01bc	4.58 ± 0.01bc	39.09 ± 4.51ab	1.45 ± 0.16a

Different lowercase letters in the same column indicate significant differences.

### 3.4 Diversity and composition of N functional genes

**Figure 4:**
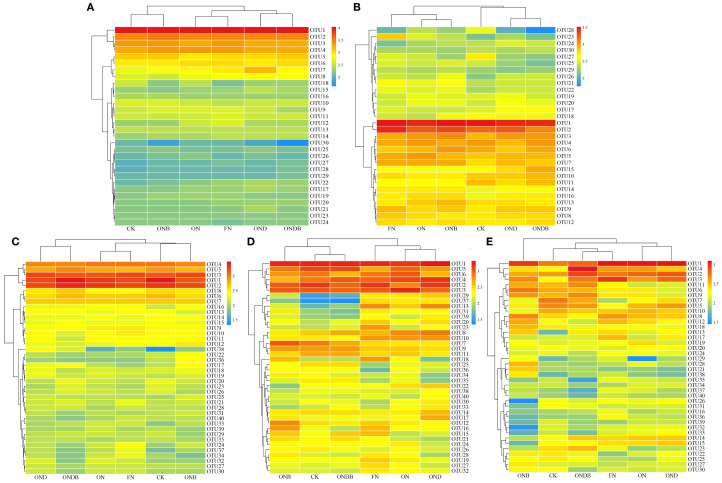
OTU clustering heatmap of AOA **(A)**, AOB **(B)**, *nirK* **(C)**, *nirS* **(D)**, *nosZ* **(E)**.

To graphically illustrate the effects of different treatments on the N cycling microbial community, hierarchical clustering analysis of OTUs was conducted across treatments (**Figure 4**). Based on the OTU clustering results for AOB, OND and ONDB were initially grouped before being clustered with CK, whereas ON and ONB were preferentially grouped and then clustered with FN. The CK, ON, and FN treatments were grouped into distinct clusters, indicating that the different N fertilizer rates significantly influenced the soil AOB community. It is noteworthy that the cluster heatmaps for the AOA, AOB, and *nirK*-containing communities revealed preferential combination of the OND and ONDB treatments (**Figures 4A–C**), indicating that DMPP application altered the composition of the *amoA*- and *nirK*-containing communities. The ON and OND treatments were initially grouped, suggesting that DMPP application alone had a minimal impact on the *nirS*- and *nosZ*-containing communities (**Figures 4D, E**). In contrast, the different N application treatments (CK, ON, FN) were grouped into separate clusters, suggesting that N application significantly influenced the *nirS*- and *nosZ*-containing communities.

**Figure 5:**
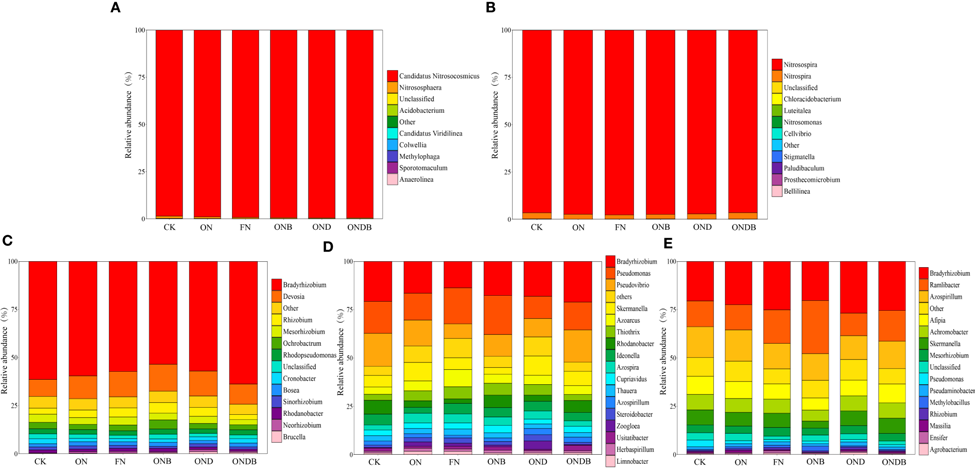
Relative abundance of AOA **(A)**, AOB **(B)**, *nirK* **(C)**, *nirS* **(D)**, *nosZ* **(E)** at genus level.

Based on the microbial communities at the genus level (**Figure 5**), the AOA community structure was relatively simple, with *Candidatus Nitrosocosmicus* identified as the dominant genus (**Figure 5A**). The AOB community was primarily composed of the genera *Nitrosospira* and *Nitrospira* at the genus level (**Figure 5B**), with *Nitrosospira* exhibiting the highest relative abundance. Application of N or biochar decreased the relative abundance of *Nitrospira*, whereas DMPP had a stimulatory effect on *Nitrospira* abundance. Notably, combined application of DMPP and biochar led to a more pronounced stimulation of *Nitrospira* abundance. The composition of the *nirK*-, *nirS*-, and *nosZ*-containing communities exhibited greater diversity at the genus level (**Figures 5C–E**). A high rate of N input or biochar application resulted in a shift of the most dominant *nirS*-containing genus from *Bradyrhizobium* to *Pseudomonas*. The most dominant genus for the *nirK*- and *nosZ*-containing communities was *Bradyrhizobium*. Biochar amendment markedly enhanced the relative abundance of *Ramlibacter* in the *nosZ*-containing community, resulting in a shift of the most dominant genus to *Ramlibacter* under the ONB treatment.

### 3.5 Relationships among N_2_O emission, soil properties, and microbial communities

**Figure 6:**
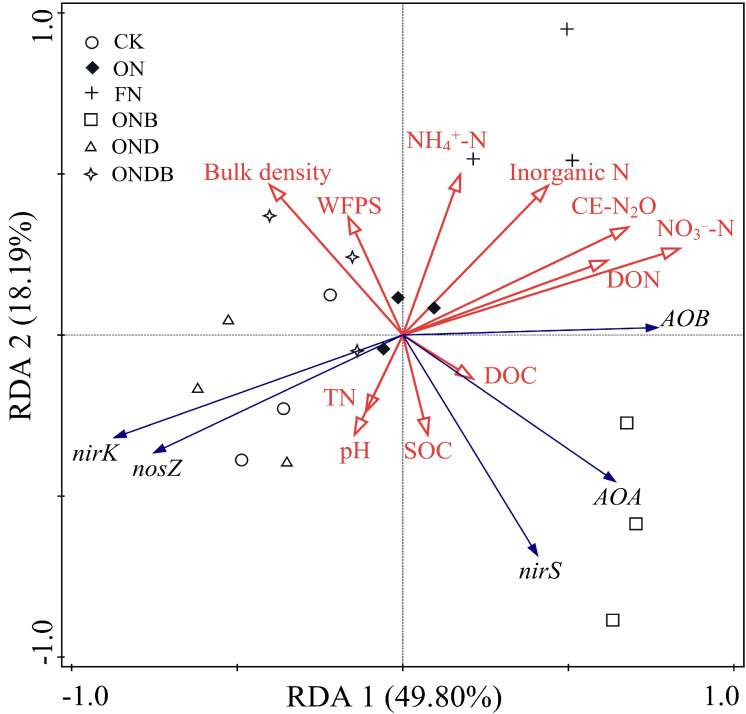
Redundancy analysis of copy number of N2O related functional genes and soil physicochemical properties.

**Figure 7:**
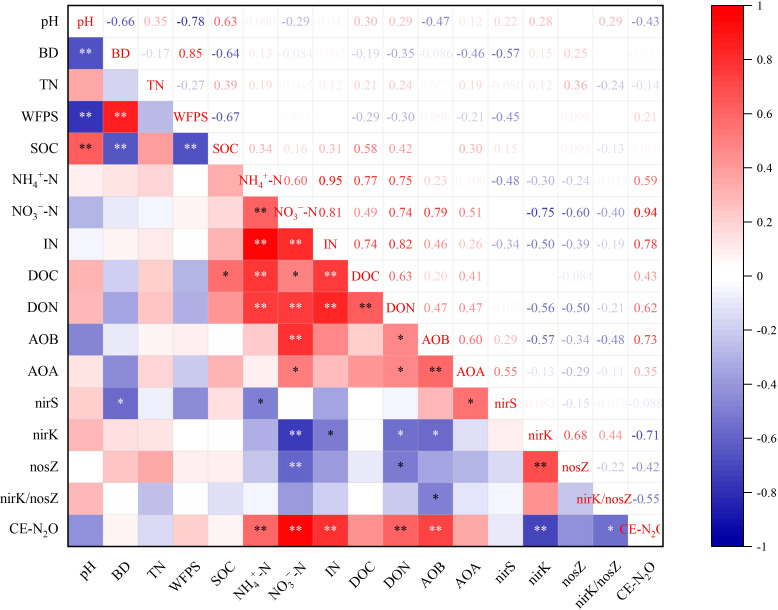
Correlation analysis of N_2_O emissions with soil properties and the abundance of N-related functional gene. * P < 0.05; **P < 0.01

**Figure 8:**
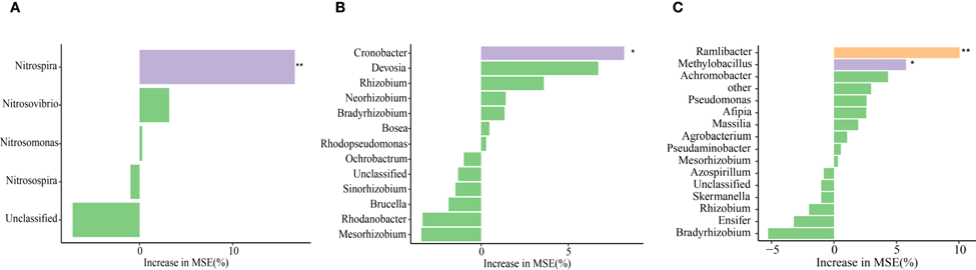
The effects of various genera of AOB **(A)**, *nirK*- **(B)** and *nosZ*- communities **(C)** on N_2_O emissions was elucidated base on random forest analysis. * P < 0.05; **P < 0.01.

Redundancy analysis was conducted to examine the inter-relationships among N_2_O emission, soil physicochemical properties, and microbial gene abundance. Axes 1 and 2 accounted for 67.99% of the total variance (**Figure 6**). The ON, ONB, and DMPP addition treatments were resolved as distinct on axis 1 (49.80%). The contents of NO_3_^−^-N and inorganic N, DOC, pH, and TN were critical indicators that influenced the experimental system. Correlation analysis indicated that NO_3_^−^-N, inorganic N, AOB, *nirK*, DON, and NH_4_^+^-N were key indicators that influenced soil N_2_O emission (**Figure 7**). Random forest analysis further revealed that *Nitrospira* was the genus within the AOB community that most significantly affected N_2_O emission, *Cronobacter* was the dominant genus responsible for N_2_O emission in the *nirK*-containing community, whereas *Ramlibater* and *Methylobacillus* in the *nosZ*-containing community were the significantly predictors of N_2_O emission (**Figure 8**).

## 4 Discussion

### 4.1 Impacts of biochar and DMPP on N_2_O emission

The N_2_O released from agricultural soils is a byproduct of nitrification and denitrification. Carbon and N play crucial roles influencing the emission of N_2_O (Cayuela et al., 2014; Li et al., 2022). Our findings indicate that the co-application of biochar and DMPP caused the most effective inhibition of N_2_O emission. Compared with the ON treatment, the ONDB treatment led to a 38% reduction in CE-N_2_O. This effect is attributed to the substantial decrease in content of the denitrification substrate (NO_3_^−^-N) under the ONDB treatment, which consequently inhibited the activity of denitrifying bacteria. The regression analysis revealed a significant negative correlation between pH and CE-N_2_O (*P < 0.01*, **Supplementary Figure S1**). The ONDB treatment significantly increased the soil pH, which may be an additional factor that contributes to the synergistic effects of biochar and DMPP in mitigating N_2_O emission. Therefore, we proposed that DMPP’s inhibition of AOB combined with biochar’s pH modulation jointly reduced N_2_O. Recent studies have demonstrated that biochar has a markedly superior capacity for N_2_O adsorption compared with soil and its mineral constituents (Xiao et al., 2018). Consequently, the ONDB treatment may enhance the adsorption and stabilization of specific N_2_O molecules within the biochar matrix. However, Li et al. (2023) reported that combination of biochar and DMPP did not lead to a significant reduction in N_2_O emissions in agricultural systems, which may be attributable to regional soil characteristics and the intrinsic properties of biochar.

Biochar application alone resulted in an increase in soil CE-N_2_O compared with that of the ON treatment. The ONB treatment significantly enhanced the soil NO_3_^−^-N content (**Table 2**), indicating that biochar incorporation significantly enhanced soil nitrification, consistent with the findings of Chen et al. (2019). Previous research has demonstrated that biochar is abundant in various volatile compounds and serves as an organic C source for denitrifying bacteria (Fu et al., 2022), thereby stimulating N_2_O emission. The present study revealed that the ONB treatment significantly increased the DOC compared with the ON treatment, thereby confirming that the incorporation of biochar (an exogenous source of organic C) enhanced soil denitrification (Weldon et al., 2019). Furthermore, biochar application significantly enhanced the SOM, thereby increasing the availability of C and N within the soil (Chagas et al., 2022). This enhancement fosters elevated diversity and activity of soil microorganisms, leading to increased oxygen consumption (Xu W. et al., 2023), and as a result, localized anoxic conditions are more conducive to denitrification.

Li et al. (2023) reported DMPP decreases N_2_O emissions by disrupting the N conversion processes within the soil, ultimately causing reduced availability of N for nitrification and denitrification. A experiment conducted by Zhao on a wheat–maize rotation system demonstrated that DMPP significantly mitigated soil N_2_O emissions, consistent with the present findings. The current study demonstrated that DMPP application resulted in a significant 32% reduction in CE-N_2_O compared with that of the ON treatment, which was largely consistent with the results of a meta-analysis of agricultural systems conducted by Ekwunife et al. (2022). Nevertheless, this reduction was markedly less than in lab experiments (Fan et al., 2019).

Fluctuations in air temperature and precipitation affect soil aeration and oxygen concentrations, which subsequently impact on N_2_O production (Yang Y. et al., 2021); in addition, the redox environment of the soil plays a critical role (Xu P. et al., 2023). In the present study, a notable increase in soil N_2_O flux emissions was observed following the second N application. Fluctuations in soil moisture, in combination with optimal surface-soil temperatures ranging from 19 to 27°C, resulted in frequent cycles of drying and wetting within the soil environment (**Supplementary Figure S2**). This dynamic created alternating conditions of oxidation and reduction, which further enhanced the denitrification process facilitated by both nitrifying and denitrifying bacteria, thereby increasing N_2_O emissions. Theodorakopoulos et al. (2017) reported that N_2_O emissions were predominantly attributable to nitrification at WFPS < 60%, and by denitrification at WFPS > 60%. The soil WFPS ranged between 17% and 71% in the present study. Consequently, it is probable that N_2_O emissions from the soil primarily originated from the nitrification pathway. Significant correlations between NO_3_^−^-N, inorganic N, NH_4_^+^-N, and CE-N_2_O were observed, which is inconsistent with the findings of Huang et al. (2019). We propose that the N_2_O emissions observed in the present study primarily originated from oxidation of soil ammonia, particularly through hydroxylamine decomposition. Furthermore, the notable positive correlation between soil DON and CE-N_2_O reinforces that DON-mediated heterotrophic ammoxidation may serve as a pivotal contributor to N_2_O production. However, the findings of this study were derived from two wheat growing seasons, and the long-term efficacy of combined biochar and DMPP application in mitigating N_2_O emissions remains to be confirmed.

### 4.2 Response of ammonia oxidizing microbial communities to N and synergist

The activity of the nitrifying bacterial community is significantly influenced by the soil environment (Zheng et al., 2019). AOA and AOB display distinct adaptations to soil NH_4_^+^ environments, with AOB predominant under elevated NH_4_^+^ conditions, whereas AOA exhibits the opposite trend (Fang et al., 2023). Previous investigations have revealed that fertilizer application markedly increases nitrifying microbial activity in the soil, leading to elevated N_2_O emissions (Li et al., 2020). The microcosmic examination of ammonia-oxidizing processes under different N fertilization regimes is rather complex, owing to the participation of a diverse array of ammonia-oxidizing microorganisms (Yang L. et al., 2021).

The present study detected a positive correlation between N_2_O emission and the abundance of AOB, which in turn increases with elevation of the N application rate. This result accords with a meta-analysis of 157 field observation datasets conducted by Ouyang et al. (2018). However, their study indicated that the abundance of AOA increases in response to N application. The present findings showed that biochar application significantly enhanced the abundance of both AOA and AOB, consistent with previous research demonstrating that biochar stimulates nitrification activity and fosters the proliferation of ammonia-oxidizing microorganisms (Xu W. et al., 2023). Application of DMPP mitigated the impact of N fertilization on the abundance of AOB. Furthermore, the synergistic effect of biochar and DMPP significantly decreased the abundance of AOA and AOB. The copy number of AOA genes was lower than that for AOB genes (AOA/AOB=0.52) at wheat harvesting (**Table 3**). These findings indicate that AOB may exhibit greater abundance and demonstrate enhanced ammoxidation activity in agricultural soils with elevated N contents (Yan et al., 2018). With regard to OTU clustering within AOB, the OND and ONDB treatments were clustered and subsequently linked with CK to form a single cluster. The ONB and ON treatments were preferentially linked before being grouped with FN to establish a distinct cluster. This finding elucidates the variation in CE-N_2_O under the different treatments. Redundancy analysis indicated that AOB was the most significant positive factor that influenced N_2_O emissions, while correlation analysis revealed that AOB contributes substantially more to N_2_O emissions than AOA.

The impact of AOB on N_2_O emissions is significantly greater than that of AOA. Further identification of specific microorganisms within AOB that modulate N_2_O emissions is warranted. Cytryn et al. (2012) amplified amoA gene fragment and revealed that the AOB community in paddy soil is predominantly composed of *Nitrosomonas*. The relative abundance of *Nitrosospira* decreased compared with *Nitrosomonas* as N application increased., as the addition of N promotes a shift from a less nutrient-rich bacterial community to a more symbiotic community. However, high-throughput sequencing revealed that the predominant genus of AOB was *Nitrosospira* in this study and that the proportion of *Nitrosospira* rose with increase in the N application rate, whereas *Nitrospira* showed an inverse relationship. Similarity, Bi et al. (2023) reported that *Nitrosospira* is the predominant ammonia-oxidizing genus in agricultural soils. Liu et al. (2021) identified *Nitrosospira* as the dominant genus in environments with high NH_4_^+^ concentrations, demonstrating an enhanced capacity for ammonia oxidation. This genus plays a crucial role in N_2_O emissions from soils characterized by high concentrations of NH_4_^+^. In the current study, the application of biochar alone (ONB) significantly enhanced the proportion of *Nitrosospira* compared with the ON treatment, while concurrently reducing *Nitrospira* abundance. However, Lin et al. (2017) demonstrated that exogenous organic C altered the AOB community, shifting from *Nitrosospira* to *Nitrosomonas*.

We propose that the primary factor contributing to this discrepancy is soil pH. In acidic conditions, the nitrification activity of *Nitrosomonas* surpasses that of Spirulina *Nitrosomonas*; however, the adaptability of *Nitrosomonas* to alkaline environmental stress is significantly lower than that of Spirulina *Nitrosomonas*. *Nitrospira* can oxidize nitrite and convert urea into ammonia, promoting the growth of nitrifying bacteria. Although its abundance remains relatively stable with increasing N application rates, it exhibits a significant correlation with N_2_O emissions (Liu et al., 2024). Likewise, in the present study, treatments that included DMPP were observed to enhance the relative abundance of *Nitrospira*, whereas the ONB and FN treatments led to a decrease in its relative abundance. Random forest analysis further demonstrated that *Nitrospira* exhibit a significant predictive capacity for N_2_O emissions. Additionally, in comparison with the ON treatment, the combination of biochar and DMPP significantly reduced the α-diversity indices (**Supplementary Table S2**), indicating that ONDB treatment significantly reduced AOB richness and diversity. Consequently, we propose that DMPP exerts an inhibitory effect on N_2_O emissions by diminishing the abundance and α-diversity of AOB, as well as by increasing the relative proportion of *Nitrospira* within the AOB community.

### 4.3 Response of denitrifying microbial communities to N and synergist

Denitrification, mediated by heterotrophic bacteria and fungi, primarily occurs in anaerobic environments, where nitrate undergoes a series of transformations (e. g. NO_2_^−^, NO, N_2_O) that ultimately yield N_2_. Previous studies have established that denitrifying bacteria harboring *nirS* and *nirK* are the primary contributors to denitrification-mediated N_2_O production, primarily because of inadequate genetic capacity for the reduction of N_2_O (Huang et al., 2019). The *nirK* and *nosZ* copy numbers under the ONB treatment were lower than those detected under the ON treatment (**Table 3**). Biochar application can foster a soil environment with an elevated C/N ratio, thereby promoting N assimilation within the soil and diminishing the availability of N substrates for denitrification (Liu Z. et al., 2021). The OTU cluster heatmaps revealed that the *nirS*-, *nirK*-, and *nosZ*-containing communities exhibited distinct responses to the various treatments. Our findings indicated that DMPP treatment (OND and ONDB) significantly altered the composition of *nirK*-containing communities (**Figure 4C**); however, DMPP application alone exhibited limited effects on the *nirS*- and *nosZ*-containing communities (**Figures 4D, E**). Conversely, biochar application alone had a pronounced impact on the *nirS*- and *nosZ*-containing communities (**Figures 4D, E**). Xiao et al. (2021) reported that application of exogenous N or C influences the soil microbial community, while the simultaneous addition may alter the dominant genera within the denitrification gene community. Similarly, in the present study, we observed a shift in the most dominant genus of *nirS*-containing community from *Bradyrhizobium* under the ON treatment to *Pseudomonas* under the FN and ONB treatments. The most dominant genus of *nosZ*-containing community transitioned from *Bradyrhizobium* to *Ramlibacter* under the ONB treatment. *Bradyrhizobium* is an aerobic azotobacter within the rhizobia order (Geddes et al., 2020), and was the most dominant genus in the *nosZ*-containing community in all treatments except for the ONB treatment. This finding emphasizes the possibility for denitrification within an aerobic environment. Both *nirK* and *nosZ* were significantly correlated with DON. The correlation coefficient between SOC and *nirS* exceeded that of SOC with the other denitrification genes (nirK, nosZ) (**Figure 7**), indicating that *nirK*- or *nosZ*-containing bacteria showed heightened sensitivity to exogenous N, whereas *nirS*-containing bacteria exhibited greater sensitivity to exogenous C compared with that of *nirK*- or *nosZ*-containing bacteria.

In this study, CE-N_2_O was significantly negatively correlated with the *nirK* gene copy number (*P < 0.01*). This finding is consistent with the conclusions by Wu et al. (2023), who established that the presence of *nirK*-containing denitrifying bacteria are critical determinants in both N_2_O consumption and production. Random forest analysis identified *Cronobacter* as a critical genus driving N_2_O emissions in *nirK*-containing communities. Similarly, Xie et al. (2024) reported that substitution with organic fertilizers affected the relative abundance of *Cronobacter* in the *nirK*-containing community, thereby mitigating N_2_O emissions. Interestingly, our findings demonstrate that despite the lack of a markedly correlation between CE-N_2_O emissions and *nosZ* gene copy numbers, while a significant negative correlation was observed with the *nirK*/*nosZ* ratio (Shi et al., 2019). Furthermore, the present findings revealed that *Ramlibater* and *Methylobacillus* in the *nosZ*-containing community exhibited a significant predictive capacity for N_2_O emissions. Consequently, the *nosZ* gene may still be among the key contributors to N_2_O emissions mediated by denitrification. Tang et al. (2024) reported that niche variations in the denitrification genes *nirK* and *nirS* resulted in differential N_2_O emissions. However, a weak correlation was observed between CE-N_2_O and *nirS* gene copy number in the present study, indicating that *nirS* was not the most critical factor influencing N_2_O emissions. Nevertheless, correlation analysis revealed a significant correlation between AOB and *nirK* copy number with N_2_O emissions; however, this does not provide direct evidence for microbiome-mediated N_2_O emission. Therefore, it is essential to conduct a more in-depth analysis in future to elucidate the contributions of various microorganisms to N_2_O emissions using microbial molecular ecology and isotope tracer methodologies.

## 5 Conclusion

Compared to the normal N application regime, delayed N application significantly reduced both CE-N_2_O and EF-N_2_O. Under the delayed N application regime, the ONB treatment increases N_2_O emissions, whereas treatment with DMPP (OND and ONDB) significantly mitigates N_2_O emissions by 32% - 38%. The CE-N_2_O exhibited a positive correlation with the copy number of AOB genes, and a negative correlation with *nirK* gene copy number and *nirK/nosZ* ratio. Random forest analysis identified that the community species of the AOB, *nirK*, and *nosZ*-containing communities are sensitive biomarkers for evaluating of N_2_O emissions in agricultural ecosystems. Consequently, the ONDB treatment is a promising strategy for mitigation of N_2_O emissions under the delayed N application regime. This approach is feasible for regions with high N inputs but requires cost-benefit analysis for farmer adoption. Future studies are encouraged to employ isotopic tracing techniques to confirm microbial pathways and conduct field trials across diverse soil types.

## Data Availability

The raw sequencing data generated from microbial diversity analyses in this study have been deposited in the Sequence Read Archive (SRA) database at NCBI under the following BioProject accession numbers: AOA (PRJNA1334209), AOB (PRJNA1334285), nirK (PRJNA1334144), nirS (PRJNA1334174), and nosZ (PRJNA1334121).
