# Supplementary material for: Combined biochar and DMPP reduce N2O emissions in wheat crops via microbial community modulation
Source: Front Plant Sci. 2025 Oct 1;16:1647453. doi: 10.3389/fpls.2025.1647453 (PMC12521238; doi:10.3389/fpls.2025.1647453)
Supplement: Supplementary file 1 [file DataSheet1.zip › Table.S1.docx]

Table S1 Primer pairs and primer sequence for real-time quantitative PCR (qPCR)

| Target gene | Primer name | Primer sequence |
| --- | --- | --- |
| AOB amoA | amoA-1F  amoA-2R | GGGGTTTCTACTGGTGGT |
| nirS | Cd3aF  R3cd | CCCCTCKGSAAAGCCTTCTTC |
| nirK-2 | Copper583F  Copper909R | GTSAACGTSAAGGARACSGG |
| Arch amoa | Arch- amoAF  Arch-amoAR | GASTTCGGRTGSGTCTTGA |
| nosz-1622 | nosZ-F  nosZ1622R | TCATGGTGCTGCCGCGYGANGG |
