# Supplementary material for: Combined biochar and DMPP reduce N2O emissions in wheat crops via microbial community modulation
Source: Front Plant Sci. 2025 Oct 1;16:1647453. doi: 10.3389/fpls.2025.1647453 (PMC12521238; doi:10.3389/fpls.2025.1647453)
Supplement: Supplementary file 1 [file DataSheet1.zip › Table.S2.docx]

Table S2 alpha diversity index of AOB

| Treatment | Chao | Shannon | Evenness |
| --- | --- | --- | --- |
| CK | 1538.73±148.73ab | 6.85±0.15ab | 0.67±0.01ab |
| ON | 1523.05±90.68ab | 7.03±0.43a | 0.68±0.03a |
| FN | 1413.52±24.23bc | 6.55±0.14bc | 0.65±0.01ab |
| ONB | 1601.41±91.05a | 7.03±0.09a | 0.68±0a |
| OND | 1408.83±81.11bc | 6.94±0.22ab | 0.68±0.02a |
| ONDB | 1327.6±74.62c | 6.39±0.11c | 0.64±0.01b |
