# Supplementary material for: Combined biochar and DMPP reduce N2O emissions in wheat crops via microbial community modulation
Source: Front Plant Sci. 2025 Oct 1;16:1647453. doi: 10.3389/fpls.2025.1647453 (PMC12521238; doi:10.3389/fpls.2025.1647453)
Supplement: Supplementary file 1 [file DataSheet1.zip › Figrue.S1.docx]

Figure. S1 linear regression relationship between soil pH and cumulative N_2_O emissions.
